# Antimicrobial photodynamic therapy with *Ligularia fischeri* extract and red light improves the restoration of *Staphylococcus aureus*-infected wounds in BALB/c mice

**DOI:** 10.3389/fcimb.2026.1827267

**Published:** 2026-05-20

**Authors:** Jin-Woo Kim, Sung-Chul Hong, Jaeyoung Kwon, Kyungsu Kang, Pahn-Shick Chang, Jin-Chul Kim

**Affiliations:** 1 Interdisciplinary Program in Agricultural Genomics, Seoul National University, Seoul, Republic of Korea; 2 Center for Natural Product Efficacy Optimization, Korea Institute of Science and Technology Gangneung Institute of Natural Products, Gangneung, Republic of Korea; 3 Department of Food Science and Biotechnology, Kunsan National University, Gunsan, Republic of Korea; 4 Center for Natural Product Systems Biology, Korea Institute of Science and Technology Gangneung Institute of Natural Products, Gangneung, Republic of Korea; 5 Department of Agricultural Biotechnology, Seoul National University, Seoul, Republic of Korea; 6 Research Institute of Agriculture and Life Sciences, Seoul National University, Seoul, Republic of Korea; 7 Center for Food and Bioconvergence, Seoul National University, Seoul, Republic of Korea; 8 Center for Agricultural Microorganism and Enzyme, Seoul National University, Seoul, Republic of Korea; 9 Natural Product Applied Science, University of Science and Technology (UST), Gangneung, Republic of Korea

**Keywords:** antimicrobial effect, *Ligularia fischeri*, photodynamic therapy, *Staphylococcus aureus*, wound infection

## Abstract

Antimicrobial photodynamic therapy is a promising approach for mitigating antimicrobial infections due to its effective skin homeostasis recovery and lower risk of inducing bacterial resistance. This study aims to investigate the ameliorative effects of combined treatment of red light and *Ligularia fischeri* extract on *Staphylococcus aureus*-infected wounds in BALB/c mice. The effects of *L. fischeri* extract (LF), red light (RL), and their combined treatment, referred to as LF-mediated antimicrobial photodynamic therapy (LF+RL), against *S. aureus* and *S. aureus*-infected wounds in BALB/c mice were determined. LF+RL demonstrated antimicrobial efficacy by reducing the number of *S. aureus* Korean Collection for Type Cultures (KCTC) 3,881 from 8.47 to 3.68 Log CFU/mL. It inhibited *S. aureus* growth in wounds, expedited wound size reduction, facilitated reepithelialization of the epidermis, and enhanced fibroplasia and collagen deposition in the dermis, mitigating *S. aureus*-induced impairment. LF+RL also up-regulated the expression of transforming growth factor-β, fibroblast growth factor-2 (FGF-2), FGF-7, and FGF-10 mRNA. These findings suggest that LF+RL improves wound healing following *S. aureus* infection. *L. fischeri* extract emerges as a potent photosensitizer responding to RL, exhibiting an anti-staphylococcal effect. LF+RL holds promise for the development of a novel therapeutic method to attenuate microbial infections.

## 1 Introduction

Chronic wounds are associated with conditions like diabetes and bacterial infection (Falanga et al., 2022; Li et al., 2022). *S. aureus* as an opportunistic pathogen could colonize in the wound and contribute to the delay of wound healing by prolonging inflammatory phase and delaying reepithelialization (Zielińska et al., 2023; Tkaczyk et al., 2022; Negm et al., 2022; De Francesco et al., 2022). While antimicrobial compounds are commonly used to prevent and treat wound infections, the rise of antibiotic-resistant pathogens poses challenges, with available antibiotics gradually decreasing (Liu et al., 2022; Hurlow and Bowler, 2022). Exploring alternative methods for accelerating infectious wound healing is crucial in substituting conventional antibiotics.

Antimicrobial photodynamic therapy (aPDT) emerges as a promising alternative for wound infection attenuation. aPDT, a noninvasive form of therapy, involves nontoxic photosensitizers (PSs) and irradiation with visible light (Yan et al., 2023; Piksa et al., 2023). Though harmless individually, the combination of a suitable light source and PS activates reactive oxygen species (ROS) in the lesion, selectively eliminating targets such as bacteria, fungi, viruses, parasites, and pathogenic tissue. Notably, aPDT poses a lower risk of bacterial resistance compared to conventional antibacterial agents (Ribeiro et al., 2022). Recently, aPDT has gained attention for its ease, cost-effectiveness, and safety in addressing infectious diseases, inflammation, and cancer (Ferreira et al., 2024; Cunha et al., 2024). The method’s effectiveness minimizes damage to adjacent tissues due to the high phototoxicity and low dark toxicity of PSs (Chen et al., 2022). While synthetic PSs effectively combat microbial infection, there are limitations to their application in aPDT, given the time-consuming chemical synthesis of single compounds and the associated risk of generating hazardous substances (Beri et al., 2020).

Edible plants present suitable sources of PSs for application in aPDT, offering advantages such as lower toxicity compared to synthetic compounds, fewer side effects, and cost-effectiveness (Afrasiabi et al., 2022; Aebisher et al., 2024). Prior research suggests that single compounds or extracts from plants serve as effective PSs, fundamental for aPDT (Kubrak et al., 2022). *Ligularia fischeri*, a perennial plant belonging to the Asteraceae family, is widely distributed in wet, shady areas, brooks, and slopes of Europe and Asia (Park et al., 2023). Traditionally cultivated for food and as a folk remedy for back pain, rheumatoid arthritis, contusions, scarlet fever, and hepatic disorders (Kim et al., 2022), recent studies have highlighted *L. fischeri*’s anti-inflammatory, hepatoprotective, and antioxidant properties (Fu et al., 2022). Natural compounds considered as PS candidates encompass curcuminoids, alkaloids, perylenequinones, anthraquinones, flavins, chlorin-type compounds, porphyrin precursors, and chlorophylls (Kubrak et al., 2022; Li et al., 2024). *L. fischeri* contains various photosensitive ingredients like terpinolene, caryophyllene, squalene, and chlorophyll (Kim et al., 2022; Mele et al., 2017; Mukherjee et al., 2022; Sofio et al., 2022; Assi et al., 2023; Ebrahimi et al., 2023). However, there is a lack of studies on *L. fischeri* as a PS applied to aPDT.

In aPDT research, evaluating the potential of natural plant extracts as PSs is essential, given their mentioned advantages. While aPDT is effective in reducing microbial infections, previous studies have primarily focused on microbial reduction, with limited exploration of the applicability of natural extracts in wound infection models. Therefore, this study aimed to assess the antimicrobial effect and the ability to mitigate wound infection using aPDT with *L. fischeri* extract (LF) and red light (RL) as a substitute for antibiotic therapy.

## 2 Methods

### 2.1 Plant materials and preparation of *L. fischeri* extract

The preparation of *L. fischeri* extract was performed by slightly modifying the method used in a previous study (Ha et al., 2023). *L. fischeri* was collected from Nochu Mountain (Gangneung, Republic of Korea). The aerial parts of the plant, including the leaves, were washed to eliminate pollutants and air-dried in the shade for a week. The entire plant material underwent extraction with absolute ethyl alcohol at 25 °C for 3 days. The raw extract was then filtered through 0.45-μm filter paper. The obtained filtrate was subsequently concentrated at 65 °C using a rotary evaporator. The resulting LF extracts were lyophilized and stored at −20 °C until further use. For experimental use, the lyophilized extract was dissolved in dimethyl sulfoxide (DMSO, Sigma-Aldrich, St. Louis, MO, USA) to prepare a stock solution (50 mg/mL). The stock solution was subsequently diluted with distilled water, PBS, or culture medium to the desired working concentrations prior to use. The final concentration of DMSO was maintained below 0.1% (v/v).

### 2.2 UV–visible absorption analysis of *L. fischeri* extract

The extract was dissolved in distilled water to a final concentration of 1 mg/mL and thoroughly mixed until fully solubilized. A volume of 100 μL of the sample was loaded into each well of a 96-well plate. The absorption spectrum in the UV–visible range (300–800 nm) was recorded using an Infinite M1000 microplate reader (TECAN Ltd., Männedorf, Switzerland). Distilled water was used as a blank, and baseline correction was performed by subtracting the absorbance of the blank from that of the sample. Each sample was measured in triplicate (n = 3), and the average values were used for analysis.

### 2.3 Bacterial strain and growth conditions

The established *S. aureus* strain (KCTC 3,881) was acquired from the Korean Collection for Type Cultures (KCTC). Bacterial growth materials were procured from BD Difco (Franklin Lake, NJ, USA). Similar to previous studies, the *S. aureus* KCTC 3,881 strain was cultured and sustained in tryptic soy broth (TSB) and tryptic soy agar (TSA) at 37 °C for 18 h (Kong et al., 2022). Activation of *S. aureus* KCTC 3,881 occurred before the experiments through three consecutive sub-cultures.

### 2.4 Antimicrobial activity

The antimicrobial activity of the combined treatment of LF and RL, referred to as LF-mediated aPDT (LF+RL), was assessed using a time-kill assay (da Rosa Pinheiro et al., 2023). RL irradiation was performed using a 660 nm Light emitting diode (LED), and its intensity was measured with an LI-250 light meter (LI-COR Biosciences, Lincoln, NE, USA). *S. aureus* KCTC 3,881 strain was diluted in TSB and adjusted to an optical density at 600 nm (OD600) of 0.8 (approximately 8–9 log CFU/mL). The diluted culture was combined with an equal volume of LF (final concentration: 20 μg/mL) and incubated in the dark for 30 min prior to RL irradiation. Subsequently, 200 μL of the culture-LF mixture was inoculated into a 96-well plate, followed by illumination with RL (660 nm, 120 W/m^2^) for 0, 5, 10, 15, 20, and 25 min. After RL irradiation, 100 μL of the mixture was extracted and serially diluted. The diluted mixtures were inoculated on TSA and incubated at 37 °C for 24 h, after which the number of viable cells was determined. Based on these results, RL was applied for 15 min in subsequent experiments. To compare the anti-staphylococcal effects of RL, LF, and LF+RL, the *S. aureus* KCTC 3,881 culture was treated with ampicillin (AMP, final concentration: 100 μg/mL), RL, LF, or LF+RL. AMP served as a positive control. The antimicrobial activity of each sample was determined. All treatments were performed under time-matched conditions with a total duration of 45 min; the RL and LF+RL groups were exposed to RL during the final 15 min (660 nm, 120 W/m²; light dose: 10.8 J/cm²), while LF and AMP groups were incubated for the full 45 min without RL irradiation. This exposure time was selected to evaluate the immediate antibacterial response under short-term treatment conditions, which is relevant for assessing rapid antimicrobial activity under conditions comparable to photodynamic treatment.

### 2.5 Bacterial reactive oxygen species measurement

Determination of the cellular accumulation of ROS was performed using DCFDA/H2DCFDA - Cellular ROS Assay Kit (ab113851, Abcam, Cambridge, United Kingdom) according to a previous study (Paul et al., 2021). *S. aureus* KCTC 3,881 strain was diluted in TSB and adjusted to an optical density at OD600 of 0.2. *S. aureus* suspension (2 mL) was mixed with DCFDA (0.1 mM, 100 μL) solution. After that, *S. aureus*-DCFDA mixture was incubated at 37 °C in the dark for 30 min. Thereafter, the DCFDA-exposed *S. aureus* were washed three times with sterile phosphate-buffered saline (PBS) to remove the unreacted dye, and suspended in PBS (2 mL). Subsequently, DCFDA-exposed *S. aureus* suspension was distributed into six experimental groups. All groups were initially incubated for 30 min following sample addition, after which an additional 15 min treatment was applied with or without RL (660 nm, 120 W/m², 15 min; light dose: 10.8 J/cm^2^) irradiation. In the first group, 100 μL of *S. aureus* suspension was mixed with 100 μL of PBS. In the second group, 100 μL of *S. aureus* suspension was mixed with 100 μL of LF (final concentration: 20 μg/mL, dissolved in PBS). In the third group, 100 μL of *S. aureus* suspension was mixed with 100 μL of PBS and irradiated with RL. In the fourth group, 100 μL of *S. aureus* suspension was mixed with 100 μL of LF (final concentration: 20 μg/mL) and irradiated with RL. In the fifth group, 100 μL of *S. aureus* suspension was mixed with 100 μL of LF (final concentration: 20 μg/mL) that had been pretreated with RL under the same irradiation conditions. In the sixth group, 100 μL of *S. aureus* suspension was mixed with 100 μL of tert-butyl hydroperoxide (100 μM, positive control). After that, the cellular accumulation of ROS was measured at 480–490 nm/527.5-542.5 nm (excitation/emission) using an Infinite M1000 microplate reader.

### 2.6 Skin cell culture and cytotoxicity determination

HaCaT cells, a human-derived keratinocyte cell line, were purchased from CLS Cell Lines Service GmbH (Eppelheim, Germany). The Human Dermal Fibroblasts (HDF) cell line was obtained from the American Type Culture Collection (ATCC, Manassas, VA, USA). HaCaT and HDF cells were seeded in Dulbecco’s Modified Eagle Medium (DMEM, Thermo Fisher Scientific, Waltham, MA, USA) containing 10% (v/v) fetal bovine serum (Thermo Fisher Scientific) and 1% (v/v) penicillin-streptomycin (Thermo Fisher Scientific), and then cultured in a 5% CO_2_ incubator at 37 °C. Cells were subcultured using 0.25% trypsin-EDTA solution (Thermo Fisher Scientific) when they reached approximately 80% confluence at 3-day intervals and used for experiments. To evaluate the cytotoxicity of the LF and LF+RL, 3-(4,5-dimethylthiazol-2-yl)-2,5−diphenyltetrazolium bromide (MTT) assay was conducted (Sukhonthasilakun et al., 2023). Cells were seeded in a 96-well culture plate at a concentration of 2 × 10^4^ cells/well and cultured for 24 h. After removing the existing medium from each well, the cultured cells were treated with 100 µL of DMEM (without phenol red, Thermo Fisher Scientific) containing the prepared LF (0–1000 µg/mL), and incubated for 30 min prior to RL irradiation, followed by RL irradiation (660 nm, 120 W/m^2^, 15 min; light dose: 10.8 J/cm^2^) or none, and then cultured for 24 h. After incubation, the medium was removed from each well, and 100 μL of MTT solution (0.5 mg/mL, Sigma-Aldrich) prepared using DMEM was added and cells were cultured for 1 h. Subsequently, the MTT solution was removed from each well, and then 100 μL of DMSO was added to dissolve the formazan crystals, and then the absorbance value was measured at 560 nm using an Infinite M1000 microplate reader.

### 2.7 Macrophage cell culture and cytotoxicity determination

RAW 264.7 cells, a murine macrophage cell line, were obtained from ATCC and maintained in DMEM supplemented with 10% (v/v) fetal bovine serum and 1% (v/v) penicillin–streptomycin in a humidified incubator with 5% CO_2_ at 37 °C. Cells were subcultured using a cell scraper at approximately 80% confluence. For cytotoxicity evaluation, cells were seeded in 96-well plates at a density of 5 × 10^4^ cells/well and incubated for 24 h. The medium was then replaced with phenol red-free DMEM containing LF (0–1000 µg/mL), and incubated for 30 min prior to RL irradiation, followed by RL irradiation (660 nm, 120 W/m^2^, 15 min; light dose: 10.8 J/cm^2^) or no irradiation, and further incubated for 24 h. Cell viability was assessed using an MTT assay, following the same procedure as described in Section 2.6.

### 2.8 *In vivo* wound healing assay

#### 2.8.1 Animal model

Seven-week-old male BALB/c mice (Orientbio, Sungnam, Republic of Korea), weighing 18–22 g, were utilized. All *in vivo* experiments adhered to the Institutional Animal Care and Use Committee of the Korea Institute of Science and Technology approval (Certification No. KIST-2021-10-126) and complied with the National Institutes of Health’s Animals policy (NIH publication No. 85-23, revised 1996). The animals were housed in a specific-pathogen-free room under controlled conditions: 22 °C ± 2 °C temperature, 50% ± 10% humidity, and a 12-h light/dark cycle. They had ad libitum access to water and standard rodent chow (LabDiet, St. Louis, MO, USA). After a week of acclimatization, a wound infection model was induced with reference to previous studies (Xu et al., 2021). The dorsal area of the mice was depilated, and they were subsequently anesthetized by injection with Avertin (Sigma-Aldrich). Then, 5 mm-diameter incisions were made on each side of the depilated dorsal area. The wounds were treated with 10 µL of PBS containing 9 Log CFU/mL of *S. aureus* KCTC 3,881 (designated as day 0) and covered with a 4.4 × 4.4-cm Tegaderm (3M, Saint Paul, MN, USA). Finally, the mice’s backs were wrapped with compression bandages to prevent contact. All mice were individually housed in separate cages to protect the wound site and to prevent aggression among mice.

#### 2.8.2 Sample treatment

After treatment with an equal volume of *S. aureus* KCTC 3,881, each group received samples on day 2. The mice were randomly categorized into 10 groups (n=6): the negative control (NC) group, PBS alone; the AMP control group, AMP alone; the LF control group, LF alone; the RL control group, PBS + RL; the LF+RL control group, LF + RL; the infection control (IC) group, *S. aureus* KCTC 3,881 alone; the AMP group, *S. aureus* KCTC 3,881 + AMP; the LF group, *S. aureus* KCTC 3,881 + LF; the RL group, *S. aureus* KCTC 3,881 + PBS + RL; the LF+RL group, *S. aureus* KCTC 3,881 + LF + RL. LF was dissolved in PBS at a concentration of 20 μg/mL and applied to the wound site at 10 μL. AMP was prepared at a concentration of 100 μg/mL and applied in the same manner. After application of PBS or LF, the wounds were incubated for 30 min to allow sufficient interaction prior to RL irradiation. All treatments were performed under time-matched conditions (total duration: 45 min). RL control, RL, and LF+RL groups were exposed to RL during the final 15 min (660 nm, 120 W/m^2^; light dose: 10.8 J/cm^2^), whereas all other groups were maintained without RL for the same duration. The wound was covered with a 4.4 × 4.4 cm Tegaderm, and photos were taken every 2 days until day 8. The wound area (mm^2^) was determined by measuring height and width with a caliper. During sample treatment, irradiation with RL, and wound area measurement, all mice were maintained under anesthesia with Avertin. On day 8, mice were euthanized, and dorsal tissue from the wounded area was harvested. The tissue was fixed in a 10% formalin solution and embedded in paraffin wax for 24 h for subsequent analysis. Tissue for mRNA and protein analysis was immediately frozen at −70 °C. Group information is listed in **Supplementary Table 1** and the experimental method is shown in **Supplementary Figure 1**.

### 2.9 Hematoxylin and Eosin staining

To determine the degree of reepithelialization of skin tissue, Hematoxylin and eosin (H&E) staining was performed (Li et al., 2023). Embedded dorsal skin tissue (5 μm) was sectioned and deparaffinized by washing three times with xylene for 3 min, then treated with 100, 95, 70, and 50% ethyl alcohol, followed by PBS. Tissues were stained with hematoxylin and eosin and observed under an optical microscope (CX43, Olympus Optical Co., Tokyo, Japan).

### 2.10 Gram staining

For Gram staining to evaluate *S. aureus* KCTC 3,881 infection (Nguyen et al., 2020), embedded tissues were deparaffinized, stained with crystal violet for 5 min, and rinsed with tap water. Tissues were incubated with Gram iodine for 2 min and washed with tap water. Subsequently, the tissues were treated with a Gram decolorizer for 30 s. After washing with tap water, the tissues were stained with safranin O solution for 1 min and dehydrated through consecutive treatments with 95% ethyl alcohol, 100% ethyl alcohol, and xylene. Each dehydration step was performed in triplicate for 3 min. Stained *S. aureus* KCTC 3,881 was observed under an optical microscope (CX43, Olympus Optical Co.).

### 2.11 Masson trichrome staining

Masson trichrome staining was conducted to determine tissue collagen content using a commercial kit (ab150686, Abcam), following the manufacturer’s instructions (Huang et al., 2023). Deparaffinized sections were fixed in Bouin’s fluid at 60 °C for 1 h, rinsed with tap water, and stained with Weigert’s iron-hematoxylin. After washing with running tap water, the sections were treated with Biebrich scarlet/acid fuchsin solution, rinsed with distilled water, and sequentially treated with phosphomolybdic/phosphotungstic acid and aniline blue solution. After treatment with 1% acetic acid, the sections were dehydrated with 95% and absolute ethyl alcohol, followed by treatment with xylene, and mounted with synthetic resin. Images were captured with a Nikon Eclipse TE2000U microscope (Nikon Corporation, Tokyo, Japan).

### 2.12 Immunohistochemistry

To determine the fibroblast content, immunohistochemistry was performed with slight modifications to a previous study (Hyuga et al., 2023; Lin et al., 2022). The sections were treated with Tris-EDTA buffer (pH 9.0) for antigen retrieval, deparaffinized, and blocked with 5% BSA (PBS containing 0.05% Tween 20, PBST) for 1 h. The sections were incubated with primary antibodies (1:200), anti-vimentin (ab92547, Abcam) or anti-alpha smooth muscle actin (α-SMA, ab32575, Abcam), for 24 h at 4 °C. After rinsing with PBST, the sections were incubated with CY3-conjugated goat anti-rabbit IgG secondary antibody (Abcam, ab6939, 1:1,000) at room temperature for 1 h. The sections were mounted with mounting medium containing DAPI (Vector Laboratories Inc, CA, USA), and digital images were captured using a Nikon Eclipse TE2000U microscope (Nikon Corporation).

### 2.13 Quantitative real-time polymerase chain reaction

Gene expression was measured using PowerUp SYBR Green Master Mix (Thermo Fisher Scientific) and a QuantStudio 6 Real-Time PCR System (Applied Biosystems, Waltham, MA, USA). Frozen dorsal tissues were cryo-grinded with a mortar and pestle in liquid nitrogen, and total RNA was isolated using an RNeasy mini kit (Qiagen, Hilden, Germany). An aliquot of total RNA (1 µg) was reverse-transcribed into cDNA using a RevertAid First Strand cDNA Synthesis Kit (Thermo Fisher Scientific). PCR mixtures (20 μL), consisting of 100 ng cDNA, PowerUp SYBR Green Master Mix (Thermo Fisher Scientific), 400 nM of each gene-specific primer, and nuclease-free water were prepared. The amplification of a single product was verified by melting curve analysis. Relative mRNA expression levels were determined using the ΔΔCq method (Livak and Schmittgen, 2001). Primers were synthesized for the following mouse genes: transforming growth factor beta (*Tgfb*), fibroblast growth factor 2 (*Fgf2*), *Fgf7*, *Fgf10*, and glyceraldehyde 3-phosphate dehydrogenase (*G3pd*). *G3pd* was used as a reference gene. All primer sequences are listed in **Table 1**.

**Table 1:** RT-qPCR primer sequences.

Transcript	Forward primer sequence (5’-3’)	Reverse primer sequence (5’-3’)
*Tgfb*	GTCACTGGAGTTGTACGGCA	AGCCCTGTATTCCGTCTCCT
*Fgf2*	AGCGGCTCTACTGCAAGAAC	GCCGTCCATCTTCCTTCATA
*Fgf7*	AGCGGAGGGGAAATGTTCG	TCCAGCCTTTCTTGGTTACTGAGA
*Fgf10*	ATTTCCCCCTGTATGCATCCTAAC	TTCCCACGGAGGCAGAACTC
*G3pd*	CATGGCCTTCCGTGTTCCTA	ACTTGGCAGGTTTCTCCAGG

### 2.14 Western blot analysis

To measure the expression level of proteins related to wound healing, western blot analysis was performed with a slight modification of the previous research method (Dorjsembe et al., 2022). Frozen dorsal tissues were cryo-grinded with a mortar and pestle in liquid nitrogen, and total protein was isolated using a T-PER™ Tissue Protein Extraction Reagent (Thermo Fisher Scientific) containing a phosphatase inhibitor cocktail and protease (Thermo Fisher Scientific). Protein concentration was determined using a BCA protein assay kit (Thermo Fisher Scientific). Appropriate amounts of protein were separated on a 10% sodium dodecyl sulfate-polyacrylamide gel and subsequently transferred onto polyvinylidene fluoride membranes. Then, the membranes were sequentially blocked with 5% Bovine serum albumin (Bioshop Canada, Ontario, Canada) solution (dissolved in Phosphate buffered saline containing 0.05% Tween 20) and probed with primary antibodies against phospho-Akt, Akt, phosphor-GSK-3β, GSK-3β, c-Myc, and α-Tubulin at 4 °C for 15 h. The membranes were incubated with appropriate secondary horseradish peroxidase (HRP)-conjugated antibodies. Each protein band of the membrane was detected and visualized using the iBright CL1000 system (Thermo Fisher Scientific) using an enhanced chemiluminescence reagent (Thermo Fisher Scientific). The antibodies used for western blotting are listed in **Supplementary Table 2**.

### 2.15 Statistical analysis

Statistical analysis was conducted using IBM SPSS version 24.0 (SPSS Inc., Chicago, IL, USA). Differences between multiple groups were analyzed using one-way analysis of variance, followed by Tukey’s *post-hoc* test (p < 0.05, p < 0.01, p < 0.001). All experiments were independently conducted at least in triplicate. Values for each experiment represent the mean of three measurements and are presented as the mean ± standard deviation.

## 3 Results

### 3.1 LF+RL exhibits antimicrobial effects against *S. aureus*

**Figure 1:**
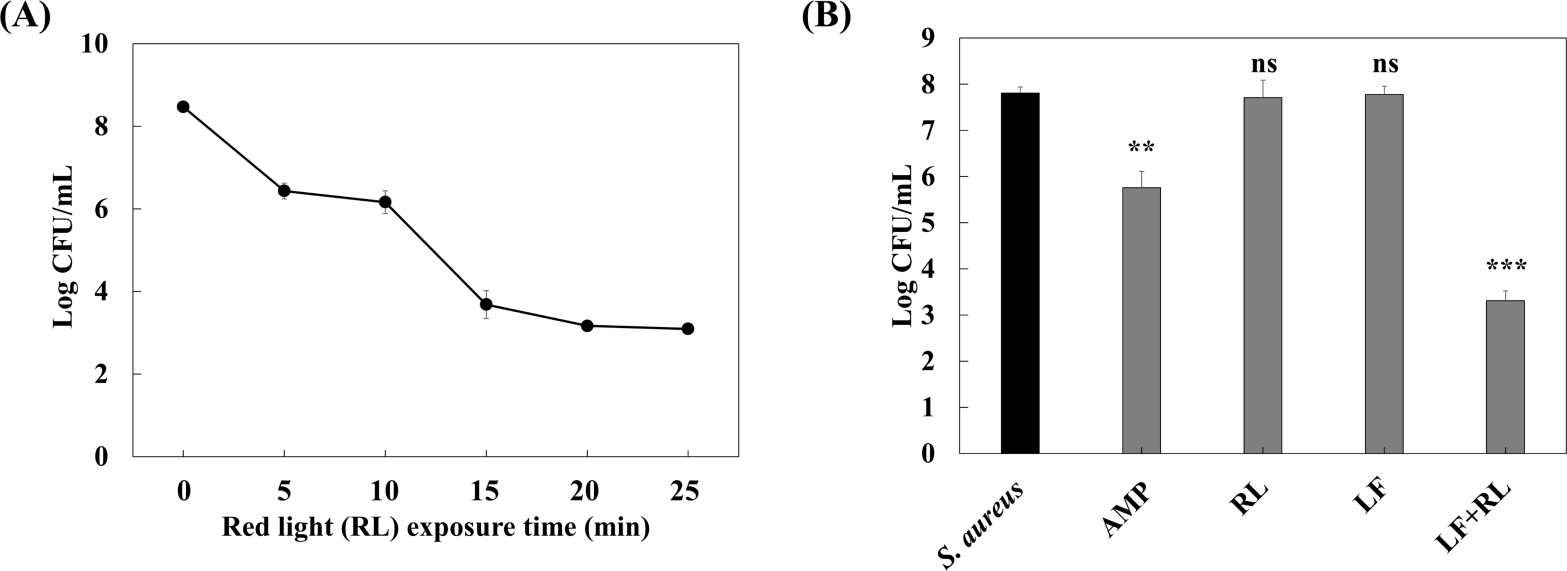
Anti-staphylococcal activity of LF+RL against *Staphylococcus aureus* KCTC 3881. **(A)** Time-kill curve of *Staphylococcus aureus* KCTC 3881 following RL irradiation at different time points in the presence of LF (LF+RL condition). **(B)** Viable cell numbers of *Staphylococcus aureus* KCTC 3881 following treatment with ampicillin, LF, RL, and LF+RL, respectively. Data are expressed as the mean ± standard deviation (n=3). ns, not significant; ^**^p < 0.01, ^***^p < 0.001 versus the *S. aureus* group. Statistical significance was analyzed by a one-way ANOVA, followed by Tukey’s *post-hoc* test.

The antimicrobial effects of LF, RL, and LF+RL against *S. aureus* were assessed through antimicrobial and time-kill assays. To determine the optimal irradiation time for LF+RL treatment*, S. aureus* cultures treated with LF were exposed to RL for varying durations. This experiment was designed to optimize irradiation time for the combined treatment rather than to evaluate the independent effects of RL or LF. **Figure 1A** illustrates the time-dependent reduction of *S. aureus* KCTC 3,881 with increasing RL irradiation time under the LF+RL condition, reaching a maximum at 15 min and remaining constant thereafter. Therefore, the observed time-dependent reduction reflects the antibacterial effect of the combined LF+RL treatment. Based on these results, a 15 min RL exposure time was selected for subsequent experiments. To ensure comparability, RL exposure was fixed at 15 min, while the total treatment duration was matched across all groups (45 min) as described in the Methods. An antimicrobial assay confirmed that neither RL nor LF alone exhibited antibacterial activity against *S. aureus* KCTC 3,881. However, LF+RL significantly reduced *S. aureus* KCTC 3,881 to approximately 3.31 Log CFU/mL (colony forming units/mL) (**Figure 1B**). Furthermore, LF+RL exhibited superior antimicrobial activity compared to AMP (positive control), which reduced *S. aureus* KCTC 3,881 to about 5.76 Log CFU/mL. To investigate the potential for photoactivation, the absorption spectrum of the extract in the UV–visible range was analyzed (**Supplementary Figure 2**). The extract exhibited characteristic absorption features, including an absorption band in the 410–420 nm region and a broad absorption band in the red region (650–700 nm), with measurable absorbance at 660 nm (ΔA = 0.28 ± 0.003), supporting its ability to be activated under the applied irradiation conditions. In addition, LF treatment alone did not upregulate intracellular ROS production in *S. aureus*. However, RL and LF+RL increased intracellular ROS levels, with the LF+RL group showing ROS levels comparable to those of the tert-butyl hydroperoxide (positive control) group (**Supplementary Figure 3**). These results suggest that the antibacterial activity of LF+RL is mediated by light-induced ROS generation.

### 3.2 Wound healing efficiency of LF+RL in the wound infection mouse model

**Figure 2:**
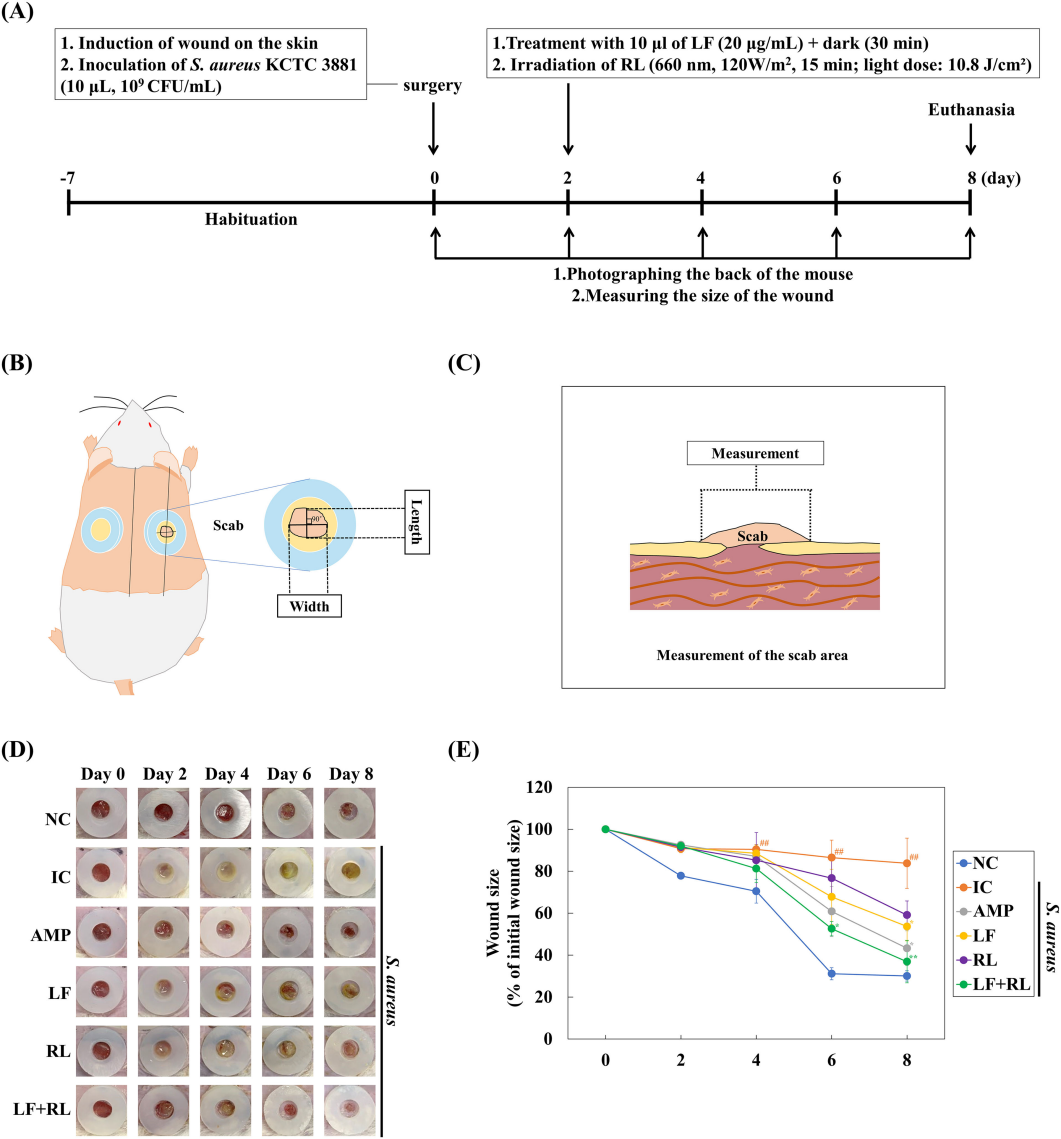
Effect of LF+RL on closure of *Staphylococcus aureus* KCTC 3881-infected wounds. **(A)** Schematic diagram of *Staphylococcus aureus* KCTC 3881 infection in the mouse splinted excisional wound model. Illustration of wound size measurement from **(B)** the top view and **(C)** the side view **(D)** Representative images of wounds in different groups during experiment (n=6). **(E)** Changes in wound size over 8 days. Data are expressed as the mean ± standard deviation (n=6). ##*p* < 0.01 versus the NC group; *p < 0.05, **p < 0.01 versus the IC group. Statistical significance is indicated at selected time points; non-significant differences are not shown for clarity. Statistical significance was analyzed by one-way ANOVA, followed by Tukey’s post-hoc test.

To determine whether the antibacterial activity of LF+RL translates into improved wound healing, its effects were evaluated in an infected wound model. Prior to the *in vivo* experiments, the cytotoxicity of LF+RL was evaluated to ensure its safe application by confirming its safety for skin and immune cells. The combined treatment conditions of LF (20 μg/mL) and RL (120 W/m², 15 min), which reduced *S. aureus*, did not exhibit cytotoxicity against HaCaT, HDF, and RAW 264.7 cells, representing epidermal, dermal, and macrophage cell types, respectively (**Supplementary Figure 4**). Based on these results, the same LF concentration and RL conditions were used for subsequent *in vivo* experiments. We evaluated the impact of LF+RL on infectious wound healing by creating wounds on the dorsal skin of mice and inoculating them with an *S. aureus* KCTC 3,881 culture solution to induce a Staphylococcal infection. The wound healing effects were evaluated across the experimental groups, including the negative control (NC), infection control (IC), ampicillin (AMP), LF, RL, and LF+RL groups, as described in Section 2.8.2. The wound healing effects of the LF, RL, and LF+RL groups were assessed (**Figures 2A–C**). Wound recovery in the LF+RL group was comparable to the level observed in the NC group (**Figure 2D**). The IC, LF, and RL groups exhibited the presence of yellow pus during wound healing and a suppression of wound recovery. To quantitatively measure the change in external wounds, we measured the scab size at the wound site. On the eighth day, the wound area in the NC group decreased to 30.07 ± 2.44% compared to the initial value (**Figure 2E**). However, in the IC group, wound healing was delayed to 83.80 ± 12.00% compared to the initial value. In the LF and RL groups, the recovery increased to 53.67 ± 6.84% and 59.10 ± 6.77%, respectively. In contrast, the AMP and LF+RL groups exhibited a reduction in wound size to 43.30 ± 8.30% and 36.92 ± 10.10%, demonstrating the same wound healing potential as the NC group (**Figure 2E**). Additionally, the AMP control, LF control, RL control, and LF+RL control groups showed no significant difference compared to the NC group, indicating that LF, RL, or LF+RL treatment did not affect acute wound healing (**Supplementary Figure 5**).

### 3.3 LF+RL promotes the skin epithelial regeneration

**Figure 3:**
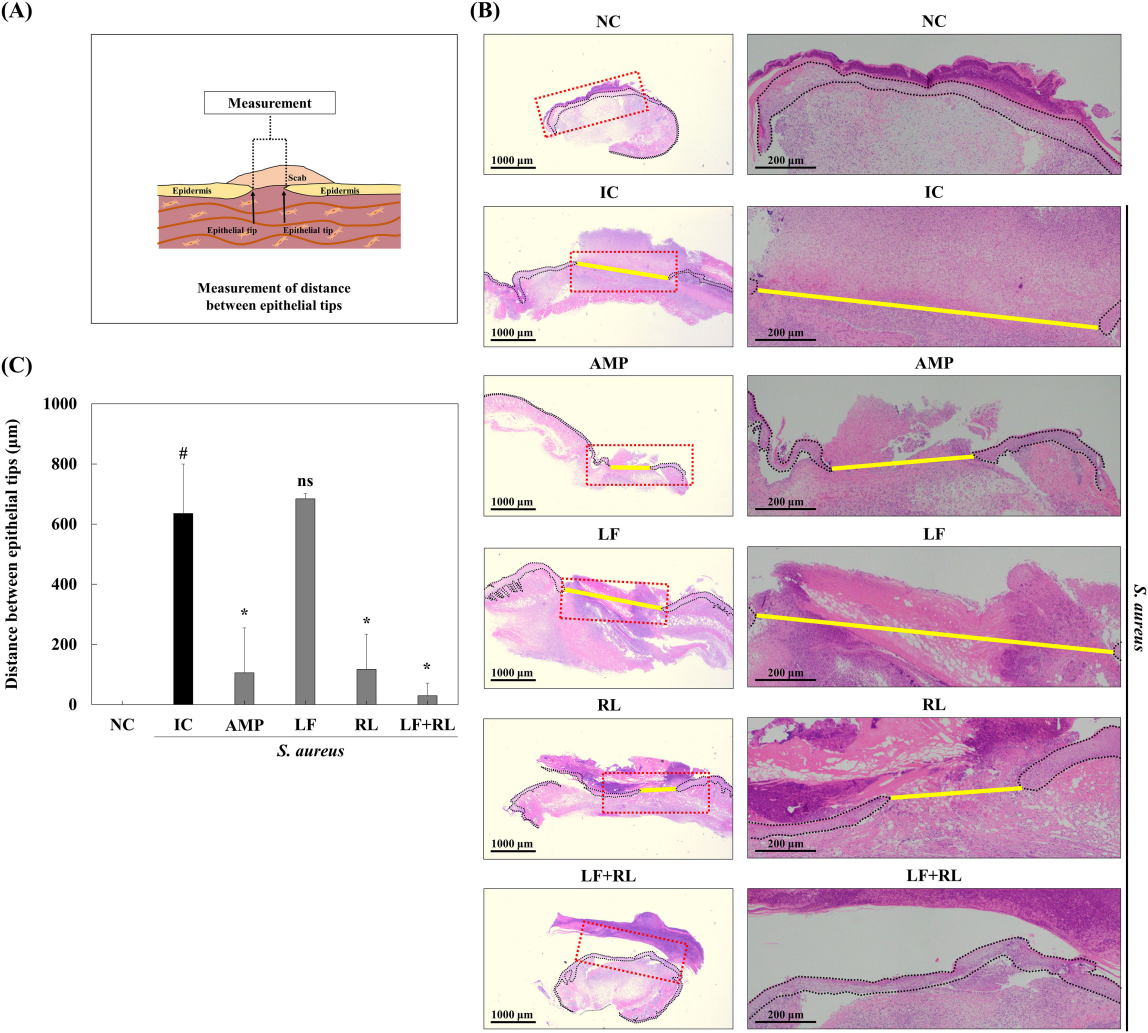
Effect of LF+RL on wound closure of *Staphylococcus aureus* KCTC 3881-infected wound on day 8. **(A)** Illustration of measuring the distance between the epithelial tips. **(B)** Representative H&E-stained images of sectioned wound tissues. The right panels show higher magnification images of the regions indicated by red dotted boxes in the corresponding low-magnification images (left panels). Yellow lines indicate the wound length between epithelial tips, and dotted lines denote the epidermal boundary (n = 6). **(C)** The distance of the epithelial tips in each wound was measured and presented as a percentage to evaluate epithelial regeneration. Data are expressed as the mean ± standard deviation (n=6). ns, not significant; ^#^*p* < 0.05 versus the NC group; ^*^p < 0.05 versus the IC group. Statistical significance was analyzed by one-way ANOVA, followed by Tukey’s *post-hoc* test.

Although wound closure was observed macroscopically, additional analysis was performed to evaluate epidermal regeneration at the tissue level. To assess epidermal regeneration at the tissue level, H&E staining was performed, and the distance between epithelial tips beneath the scab was measured (**Figure 3A**). The results showed that the internal wound tissue in the NC group was completely healed by the eighth day (**Figures 3B, C**). In contrast, among the groups infected with *S. aureus* KCTC 3,881, the rate of epithelial recovery was most significant in the LF+RL group compared to the IC, AMP, LF, and RL groups (**Figures 3B, C**). Meanwhile, reepithelialization in the RL group was markedly high, suggesting that RL exposure may contribute to increasing the tissue recovery activity of skin epithelial cells, which is vital for wound healing.

### 3.4 LF+RL exhibits anti-staphylococcal effects in the wound

**Figure 4:**
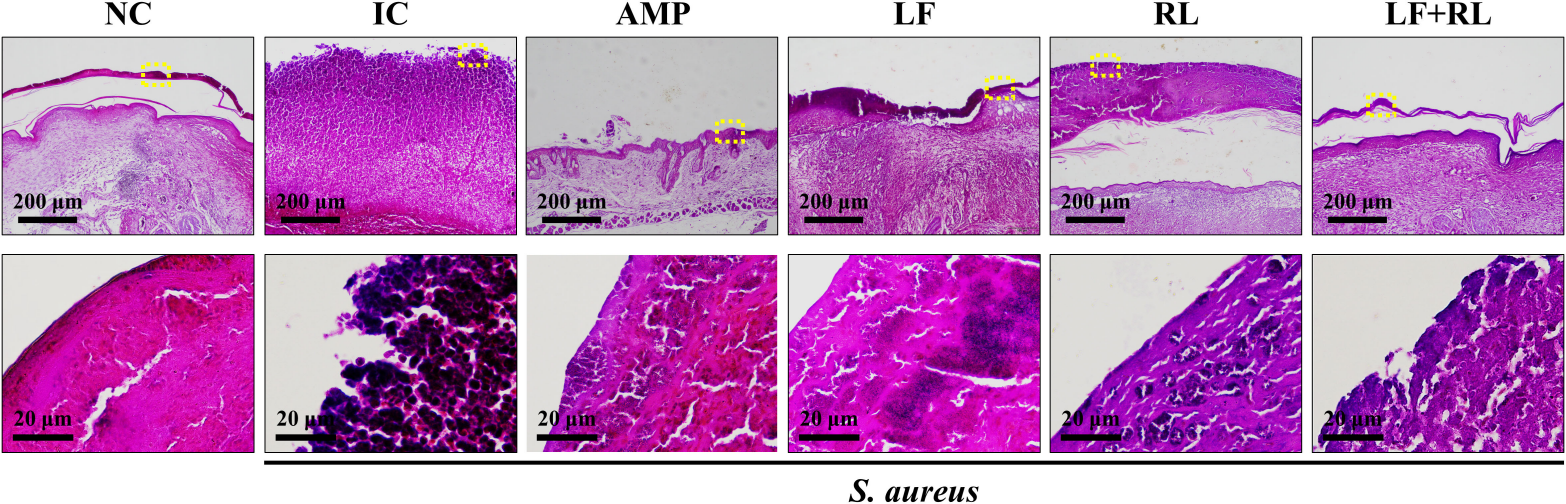
LF+RL exhibits antimicrobial effects on *Staphylococcus aureus*-infected wounds. Representative Gram-stained images of wound tissues. The lower panels show higher magnification images of the regions indicated by yellow dotted boxes in the corresponding low-magnification images (upper panels). Yellow circles highlight representative bacterial aggregates (n = 6).

To confirm the penetration of *S. aureus* into the skin, Gram staining was applied to the wound site. Bacteria were not detected in the NC group, while stained bacteria were observed in the IC, LF, and RL groups (**Figure 4**). In contrast, the AMP and LF+RL groups showed markedly reduced Gram staining, with bacterial signals primarily confined to limited regions at the wound surface, indicating effective suppression of *S. aureus* KCTC 3,881. These findings are consistent with the improved wound healing observed in the LF+RL group. This pattern suggests that LF+RL effectively reduced bacterial burden and restricted bacterial distribution, with signals largely confined to the wound surface and minimal extension into deeper tissue layers.

### 3.5 LF+RL promotes the collagen deposition and fibroplasia in dermis

**Figure 5:**
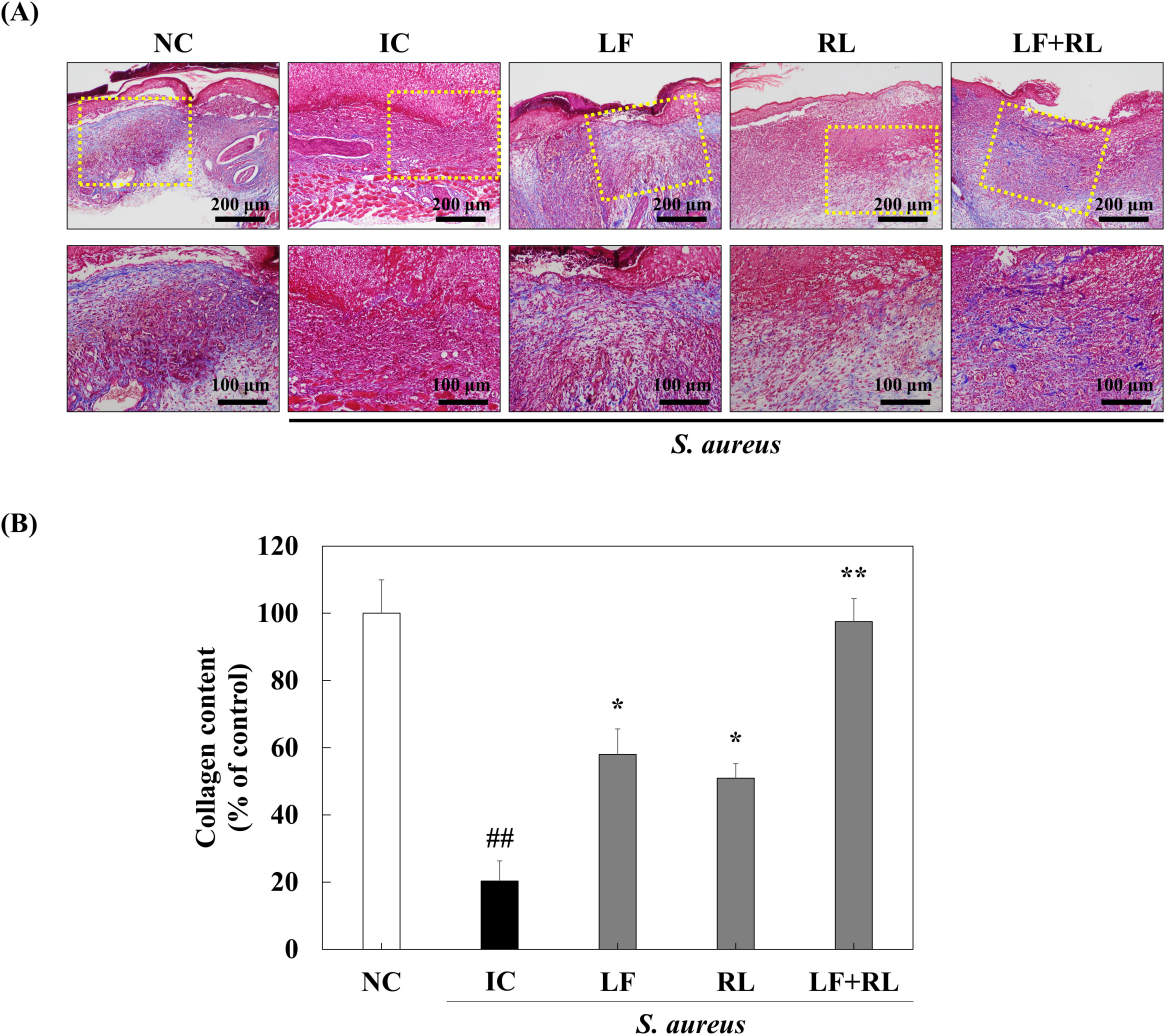
LF+RL promotes collagen biosynthesis in *Staphylococcus aureus*-infected wounds. **(A)** Representative Masson trichrome stained images of wound tissues on day 8, showing collagen deposition (blue). The lower panels show higher magnification images of the regions indicated by yellow dotted boxes in the corresponding low-magnification images (upper panels) (n = 6). **(B)** The collagen content was quantified using Image J software. Data are expressed as the mean ± standard deviation (n=6). ns, not significant; ^##^*p* < 0.01 versus the NC group; ^*^p < 0.05, ^**^p < 0.01 versus the IC group. Statistical significance was analyzed by a one-way ANOVA, followed by Tukey’s *post-hoc* test.

**Figure 6:**
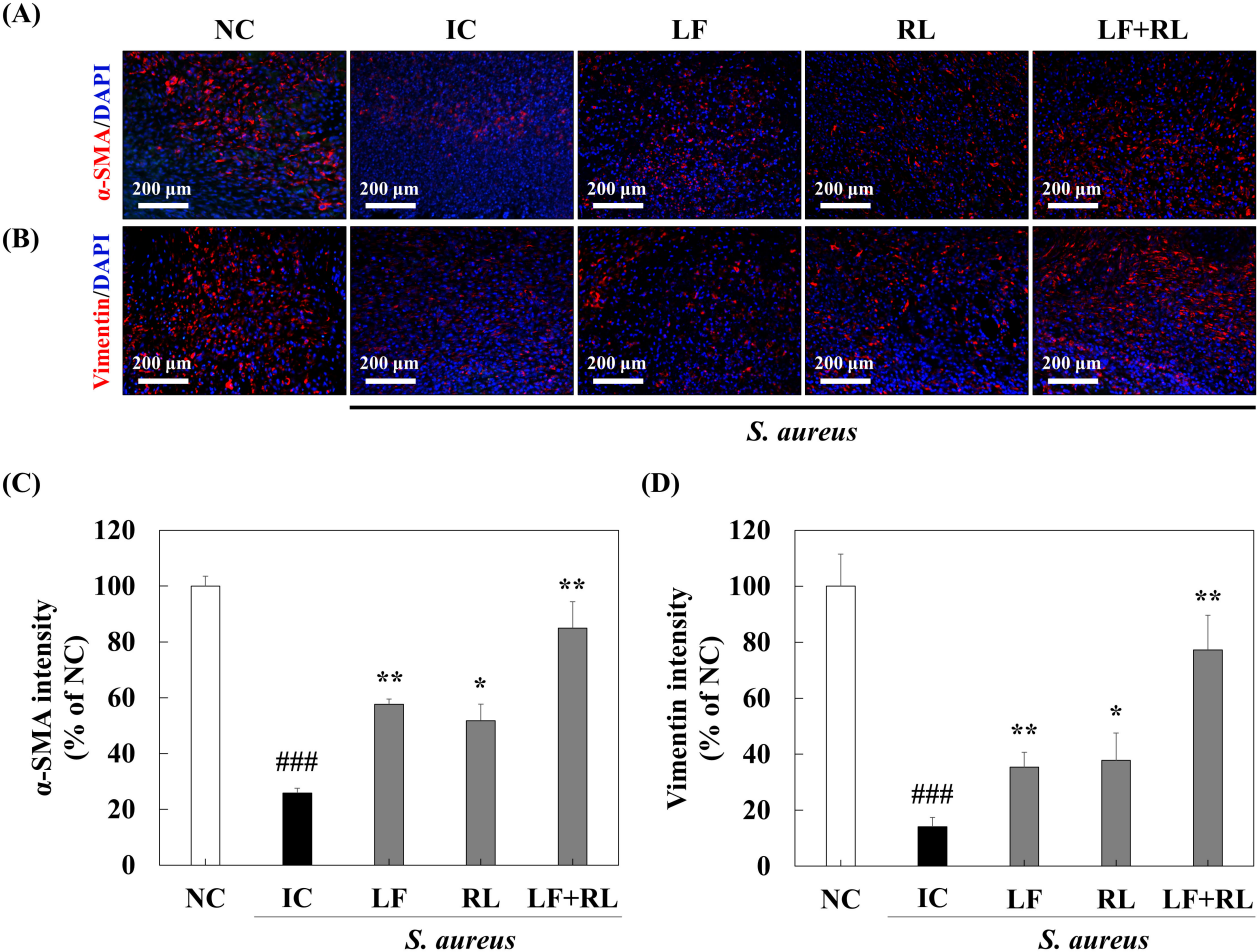
LF+RL promotes fibroplasia in the *Staphylococcus aureus*-infected wound. **(A)** Representative immunohistochemical staining of α-SMA and **(B)** vimentin to evaluate fibroblast content on day 8 (n = 6). Relative intensity of **(C)** α-SMA and **(D)** vimentin. Data are expressed as the mean ± standard deviation (n=6). ns, not significant; ^###^*p* < 0.001 versus the NC group; ^*^p < 0.05, ^**^p < 0.01 versus the IC group. Statistical significance was analyzed by a one-way ANOVA, followed by Tukey’s *post-hoc* test.

Masson trichrome staining and immunohistochemistry were conducted to evaluate the healing effect of LF+RL within the dermis. The collagen content was highest in the NC group and lowest in the IC group. In contrast to the IC group, collagen content increased in the LF and RL groups. Additionally, the LF+RL group exhibited similar levels of collagen content and deposition compared to the NC group (**Figure 5**), indicating enhanced dermal tissue reconstruction following LF+RL treatment. In addition, the fibroblast markers α-SMA, and vimentin were highest in the NC group and lowest in the IC group. The intensity increased in the LF and RL groups. Furthermore, in the LF+RL group, α-SMA and vimentin intensity increased compared to the IC group (**Figure 6**), suggesting that LF+RL promotes fibroblast activation and contributes to dermal regeneration.

### 3.6 LF+RL improves expression of markers associated with skin regeneration

**Figure 7:**
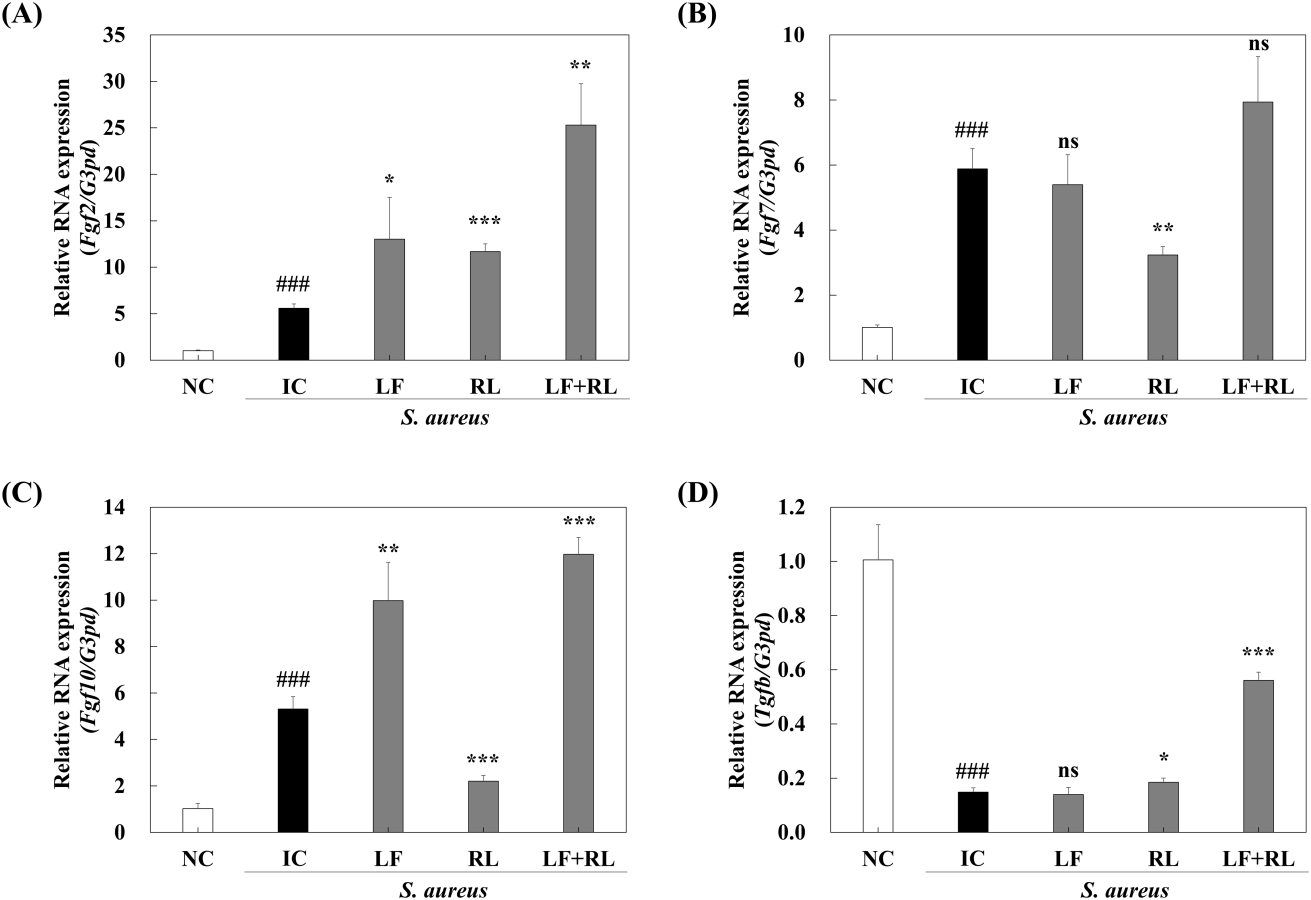
LF+RL improves *Staphylococcus aureus*-infected wounds by modulating transcriptional expression. The mRNA expression levels were measured by qRT-PCR analysis. Relative mRNA expression levels of **(A)** *Fgf2*, **(B)** *Fgf7*, **(C)** *Fgf10*, and **(D)** *Tgfb*. Data are expressed as the mean ± standard deviation (n=6). ns, not significant; ^###^*p* < 0.001 versus the NC group; ^*^p < 0.05, ^**^p < 0.01, and ^***^p < 0.001 versus the IC group. Statistical significance was analyzed by a one-way ANOVA, followed by Tukey’s *post-hoc* test.

To further investigate the mechanism underlying LF+RL-mediated skin regeneration, the expression of TGF-β, FGF-2, FGF-7, and FGF-10 mRNA in wound tissues was analyzed. The expression of these genes is related to the regeneration of the epidermis and dermis. The NC group had relatively low expression levels of FGF-2, FGF-7, and FGF-10 mRNA among the experimental groups. The LF+RL group showed the highest expression levels of FGF-2, FGF-7, and FGF-10 mRNA compared to the *S. aureus*-inoculated groups (**Figures 7A–C**). In contrast to the NC group, the expression of TGF-β decreased in the IC group and remained unchanged in the LF and RL groups; however, the LF+RL group exhibited an increase in TGF-β mRNA expression level (**Figure 7D**). In addition, **Supplementary Figures 6**, **7** showed that *S. aureus* infection upregulated phosphorylation of Akt, GSK-3β, and expression of c-Myc. LF treatment alone did not affect the expression level of these proteins in infected wounds. However, RL and LF+RL downregulated phosphorylation of Akt, GSK-3β, and expression of c-Myc. Especially, the expression level of c-Myc in the LF+RL group was equivalent to that of the NC group.

## 4 Discussion

In this study, we assessed the antibacterial activity of LF+RL for aPDT and its potential therapeutic application for improving wound infections. aPDT could serve as a substitute method for alleviating microbial infections, offering advantages such as nontoxicity to the skin and minimal occurrence of microbial resistance. RL (600–700 nm) is commonly employed as a light source for aPDT due to its nontoxic nature, deep tissue penetration (Mahmoudi et al., 2018), and applicability in treating conditions such as hyperpigmentation, acne, aging, wrinkles, and burns (Kim et al., 2023; He et al., 2024). *L. fischeri* is widely utilized in Europe and East Asia as a food and medicinal plant with various bioactivities. LF+RL may present an alternative method for enhancing wound healing by reducing bacterial infection.

*S. aureus* is one of the most resistant clinical pathogens and a significant risk factor for wound infection (Rasmi et al., 2022). We evaluated the antimicrobial effect of LF+RL against *S. aureus* KCTC 3,881. The antibacterial activity of LF-mediated aPDT increased with irradiation time up to 15 min, after which no further enhancement was observed, indicating an optimal exposure duration. In addition, neither LF nor RL alone exhibited antibacterial activity, whereas their combination resulted in a significant reduction in bacterial viability, demonstrating a clear synergistic effect. Pre-irradiation of LF did not enhance intracellular ROS production, suggesting that ROS generation depends on the simultaneous interaction between LF and light rather than prior light exposure. In contrast, the combined LF+RL treatment markedly increased intracellular ROS levels, supporting its role in photoinduced antibacterial activity. Therefore, the antibacterial activity of LF+RL is likely associated with the increase in intracellular ROS production in *S. aureus* KCTC 3,881, and these results suggest that LF may act as a potential photosensitizer under RL irradiation. To further support the proposed photoactivation mechanism, the absorption spectrum of the extract exhibited characteristic absorption features, including an absorption band in the 410–420 nm region and a broad absorption band in the red region (650–700 nm). These spectral characteristics indicate that the extract contains photoactive compounds capable of absorbing red light under the applied irradiation conditions. In a previous study (Ha et al., 2023), *L. fischeri* extract was reported to contain pheophorbide-related compounds, which belong to the porphyrin family and are well-known PSs (Kou et al., 2017). Porphyrin-based compounds typically exhibit absorption bands in both the 400–420 nm region and the red region (600–700 nm) (Montaseri et al., 2020; Wang et al., 2022a; Yin et al., 2024), which is consistent with the spectral features observed in this study. These compounds are known to generate ROS upon light activation, suggesting that similar constituents in LF are likely responsible for the enhanced ROS production and antibacterial effects observed in this study.

*S. aureus* causes opportunistic infections in weakened immune systems, such as wounds (Guidi et al., 2020; Mary et al., 2021), disrupting the skin structure that serves as a physical barrier against pathogens. In wounds, *S. aureus* can form biofilms and delay wound healing (Papadopoulou et al., 2023). In the wound infection model established by inoculating *S. aureus*, the infection control group exhibited pus formation and delayed wound closure, indicating impaired healing due to bacterial infection. LF or RL alone treatment exhibited no elimination of *S. aureus* KCTC 3,881 in the wound; however, the LF+RL group exhibited a marked reduction of *S. aureus* KCTC 3,881 in the wound among *S. aureus*-infected groups. *S. aureus* can penetrate the skin layers, including the epidermis and dermis, and impair wound healing by delaying reepithelialization and collagen synthesis (Nakatsuji et al., 2016; Breuer et al., 2002; Bhattacharya and Horswill, 2024). Therefore, histopathological alterations in the wound tissue are critical indicators of infection-associated healing impairment. Reepithelialization decreased in the IC group, but increased in the LF+RL group. The RL group showed an ameliorated reepithelialization rate, indicating that the skin regeneration effect of RL itself contributed (Erdle et al., 2008). LF+RL markedly reduced bacterial presence in the wound, whereas in the infection control group, strong Gram staining was observed at the wound surface and gradually decreased toward deeper tissue layers, forming a depth-dependent gradient, indicating that bacteria were predominantly concentrated near the surface but also penetrated into the underlying tissue. Although LF or RL alone partially improved dermal regeneration, the combined LF+RL treatment resulted in a more pronounced recovery of dermal structure, indicating an enhanced therapeutic effect. These results suggest that the reduction of *S. aureus* KCTC 3,881 may contribute to these effects.

The wound healing process involves four phases that occur in a precise and regulated manner: hemostasis phase, inflammation phase, proliferation phase, and remodeling phase (Wang et al., 2022b). Prolongation or interruptions in the process, due to causes such as bacterial infection leading to the prolongation of the inflammation phase, could delay wound healing. The NC group appeared to have progressed to the remodeling phase of wound healing, as indicated by the restoration of epidermal and dermal structures. Compared to the LF+RL group, other treatment groups exhibited delayed progression of the wound healing process. Notably, LF+RL markedly upregulated the expression of FGF-2, FGF-7, and FGF-10, which are key regulators of epidermal and dermal regeneration. As shown in other studies, the reepithelialization of the skin cells progressed more rapidly when the expression levels of FGF-7 and FGF-10 were increased (Takaya et al., 2022; Tsuboi et al., 1993; Werner and Grose, 2003). During wound healing, FGF-2 promotes angiogenesis and accelerates both the epithelial-mesenchymal transition in the epidermis and granulation tissue formation and matrix deposition in the dermis (Syromiatnikova et al., 2024; Yao et al., 2024). Thus, it is estimated that the eradication of *S. aureus* KCTC 3,881 in the wound increased reepithelialization in the epidermis and remodeling in the dermis. The NC group exhibited relatively low mRNA expression levels of FGF-2, FGF-7, and FGF-10, which may indicate that the epidermal layer recovered to some extent. TGF-β expression was reduced in the infection control group, whereas LF+RL treatment restored its expression to higher levels compared to infected groups. In the remodeling phase, TGF-β inhibits the synthesis of matrix metalloproteases, which could degrade the extracellular matrix in the dermis, leading to the accumulation of collagen fibers (Menke et al., 2008; Sabino and Keller, 2015). Moreover, the expression of TGF-β is noticeably down-regulated in chronic wounds (Liarte et al., 2020). Thereby, it is suggested that the mRNA expression of TGF-β and FGFs in the wound site is altered by eliminating *S. aureus*. In addition, c-Myc is overexpressed in chronic wounds, which inhibits the reepithelialization of wound tissue (Zhang et al., 2021). In wounds infected with *S. aureus* KCTC 3,881, c-Myc was overexpressed. Meanwhile, c-Myc expression reduced in the LF+RL group similarly to that of the NC group. Since TGF-β inhibits c-Myc expression (Frederick et al., 2004), these results support that the promotion of wound healing by LF+RL is due to increased TGF-β expression by eliminating *S. aureus*. In addition, it is known that the Akt/GSK-3β pathway is activated in chronic wound (Jere et al., 2023). It induces inflammatory responses such as activation of immune cells and plays an important role in immune function (Guerau-de-Arellano et al., 2022; Fu et al., 2025). On the other hand, activation of Akt could inhibit GSK-3β and lead to increased c-Myc expression (Thongsom et al., 2023). Supporting protein expression patterns further suggest the involvement of this mechanism. These results suggest a possible involvement of the Akt/GSK-3β pathway in the wound healing process. Since this pathway could be regulated by various factors such as FGFs (Wang et al., 2022c), further study is necessary to elucidate the relationship between wound infection by *S. aureus* and the Akt/GSK-3β pathway.

Although the present findings support the potential of LF as a photoactive material, further studies are needed to determine whether specific active compounds in LF function to alleviate wound infection. In addition, quantitative assessment of *in vivo* bacterial burden and the inclusion of clinically relevant positive controls would further strengthen the validation of the antibacterial efficacy and therapeutic potential of LF+RL treatment. Our study focused on the extract itself due to its advantages, including cost-effectiveness, time efficiency, and reduced waste in preparation, which are beneficial for therapeutic applications. This study aimed to preliminarily assess the applicability of aPDT for infectious wound treatment. Meanwhile, wound infection is one of the most common problems in surgical operations and is a major cause of fatality and morbidity in patients (Saleem et al., 2019). In particular, complex wounds such as chronic diabetic ulcers and burns are susceptible to microbial infection. A major feature of diabetes is reduced blood flow, which suppresses the function of immune cells essential for fighting infection, and hyperglycemia can create an environment favorable for microbial proliferation (Mahmoud et al., 2024; Sayampanathan, 2016). In addition, burns destroy the skin, a barrier to pathogen infection, and could induce systemic inflammatory responses and immunosuppression, further increasing the risk of infection (Roy et al., 2024). Therefore, effective control of microorganisms is essential not only for surgical wound treatment, but also for wound healing with a high risk of microbial infection, such as diabetic ulcers and burns. Generally, wound infection is suppressed by antibiotics, but since the use of antibiotics has some disadvantages, such as the occurrence of resistance (Sisay et al., 2019), it is expected that a combination of photodynamic therapy and natural products might be an effective alternative.

## 5 Conclusions

In conclusion, LF+RL has low cytotoxicity against skin cells and does not markedly affect normal wound healing, suggesting that its beneficial effects are primarily attributable to its antibacterial activity. LF+RL exhibited antimicrobial effects against *S. aureus* and mitigated the *S. aureus*-induced inhibition of wound healing. These findings suggest that aPDT, with the combination of LF and RL, may be a substitute for antibiotics in wound infections. Although further studies are necessary to determine which compound of LF functions as a PS with antimicrobial activity using RL, our results indicate that the combination of LF and RL prevents the delay of wound healing caused by bacterial infection. Taken together, the application of aPDT with a plant extract is a promising strategy for the development of a novel method to restore wound infection.

## Data Availability

The original contributions presented in the study are included in the article/**Supplementary Material**. Further inquiries can be directed to the corresponding authors.
