# Supplementary material for: Antimicrobial photodynamic therapy with Ligularia fischeri extract and red light improves the restoration of Staphylococcus aureus-infected wounds in BALB/c mice
Source: Front Cell Infect Microbiol. 2026 May 20;16:1827267. doi: 10.3389/fcimb.2026.1827267 (PMC13230172; doi:10.3389/fcimb.2026.1827267)
Supplement: Supplementary file 1 [file Table1.docx]

Supplementary Material

**TABLE S1** Group information used in the wound infection mouse model experiment

| **Group** | **Wound infection^1^** | **PBS^2^** | **AMP^3^** | **LF^4^** | **RL^5^** |
| --- | --- | --- | --- | --- | --- |
| NC | - | + | - | - | - |
| AMP control | - | - | + | - | - |
| LF control | - | - | - | + | - |
| RL control | - | + | - | - | + |
| LF+RL control | - | - | - | + | + |
| IC | + | + | - | - | - |
| AMP | + | - | + | - | - |
| LF | + | - | - | + | - |
| RL | + | + | - | - | + |
| LF+RL | + | - | - | + | + |

1, Wound inoculated with *S. aureus* KCTC 3,881 (9 Log CFU/mL, 10 µL) two days before sample treatment; 2, PBS (10 μL) treatment; 3, Ampicillin (100 μg/mL, 10 μL) treatment; 4, *L. fischeri* extract (20 μg/mL, 10 μL) treatment; 5, red light (660 nm, 120 W/m^2^) irradiation for 15 min (light dose: 10.8 J/cm²).

**TABLE S2** Antibodies for western blot analysis

| **Target** | **Type** | **Dilution rate** | **Brand** | **Cat. No.** |
| --- | --- | --- | --- | --- |
| phospho-Akt | Rabbit IgG (primary) | 1:1000 | CST^1^ | #4060S |
| Akt | Rabbit IgG (primary) | 1:1000 | CST | #4685S |
| phospho-GSK-3β | Rabbit IgG (primary) | 1:1000 | CST | #5558S |
| GSK-3β | Rabbit IgG (primary) | 1:1000 | CST | #12456S |
| c-Myc | Rabbit IgG (primary) | 1:1000 | CST | #9402S |
| α-Tubulin | Rabbit IgG (primary) | 1:2000 | CST | #2144S |
| Rabbit IgG^2^ | Mouse IgG-HRP^3^ (secondary) | 1:10000 | SCBT^4^ | #SC-2357 |

1, Cell Signaling Technology (Danvers, MS, USA); 2, IgG, immunoglobulin G; 3, HRP, horseradish peroxidase; 4, Santa Cruz Biotechnology (Dallas, TX, USA).


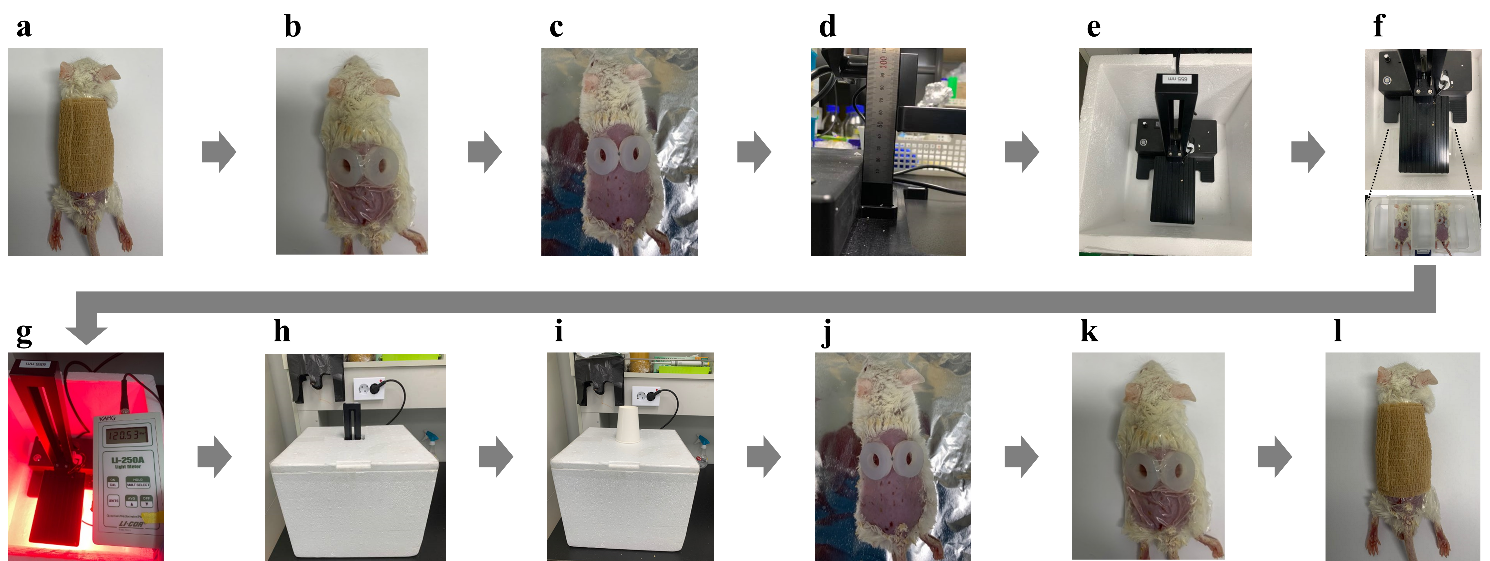


**FIGURE S1.** Induction of wound infection with *Staphylococcus aureus* and treatment with LF+RL. LF+RL treatment sequence, a: Release the pressure bandage covering the mouse's body, b: Remove the tegaderm from the mouse's back, c: Mice were anesthetized with Avertin prior to dispensing 10 μL of LF (20 μg/mL) into the wounded area on the back, followed by incubation in the dark for 30 min, d: Adjust the height of the LED lamp and the area to be treated with the LED to be 45 cm, e: Place the LED lamp in a sealed box measuring 32.5 cm in width, 32.5 cm in length, and 25.5 cm in height, f: e: Place the mice in a partition so that they can be positioned individually, and set a section where the LED lamp's irradiance can be directly shined on, g: Set the LED irradiance to 120 W/m^2^, h: Close the lid of the box, i: Block the area where the light from the LED lamp leaks out and wait for 15 minutes, j: Take the mouse out of the box, k: Cover the wounded area of ​​the mouse with tegaderm, l: Wrap the mouse's torso with a pressure bandage.


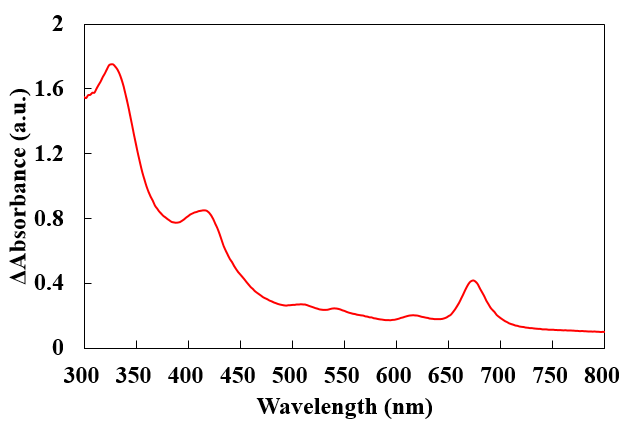


**FIGURE S2.** UV–visible absorption spectrum of LF. LF (1 mg/mL in distilled water) was analyzed over a wavelength range of 300–800 nm. Distilled water was used as a blank, and absorbance values were baseline-corrected (ΔAbsorbance). Data are presented as mean values (n = 3), and error bars are omitted for clarity.


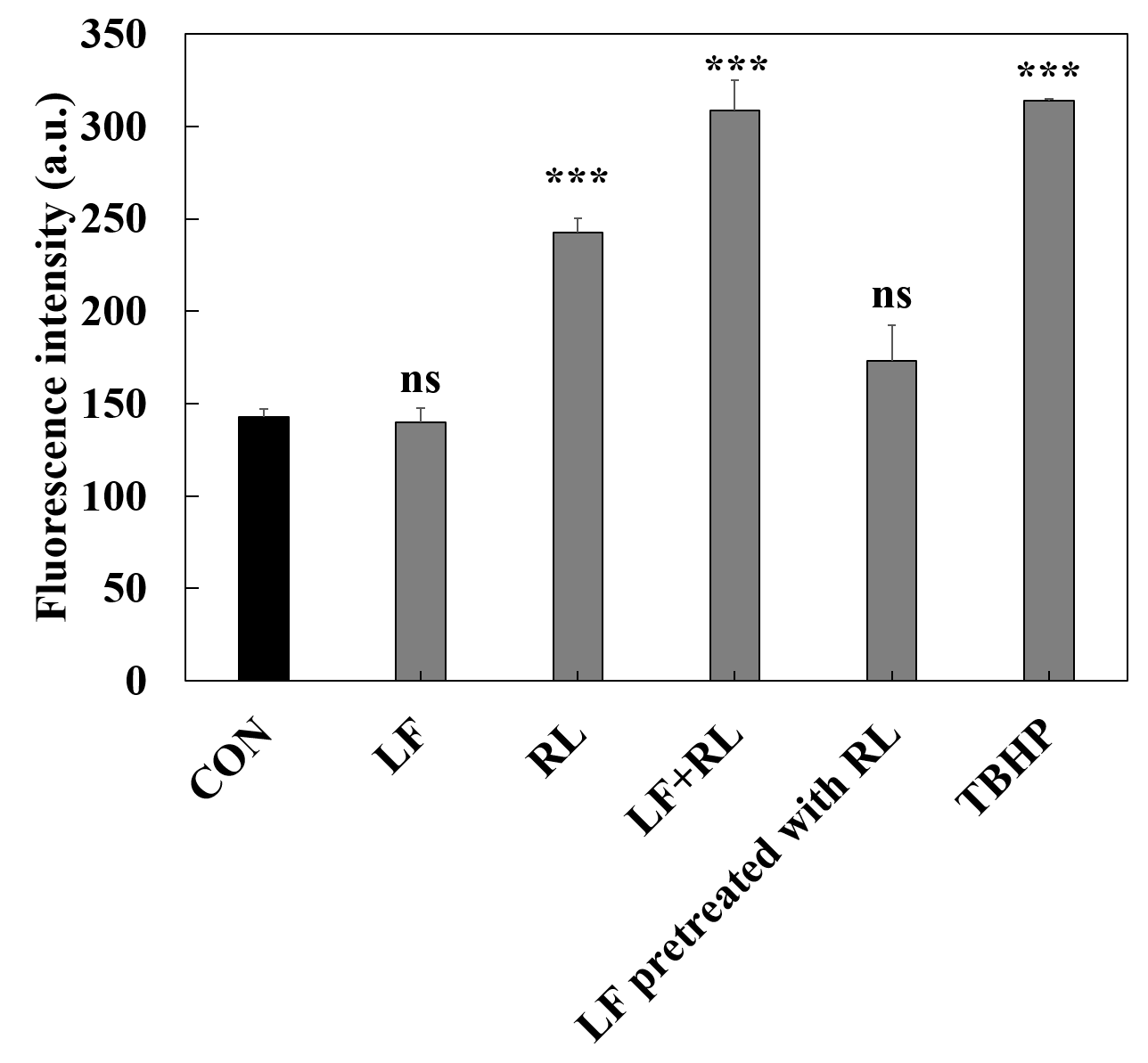


**FIGURE S3.** LF+RL induces intracellular ROS production in *S. aureus* bacterial cells. Quantification of intracellular ROS production in bacterial cells using the H_2_DCFDA assay. Data are presented as mean ± SD (n = 3). ns, not significant; ^*^*p* < 0.05, ^**^*p* < 0.01, and ^***^*p* < 0.001 versus CON. Statistical significance was analyzed by a one-way ANOVA, followed by Tukey’s post-hoc test. CON: None treated group, LF: LF treated group, RL: RL treated group, LF+RL: LF and RL treated group, TBHP: tert-butyl hydrogen peroxide treated group.


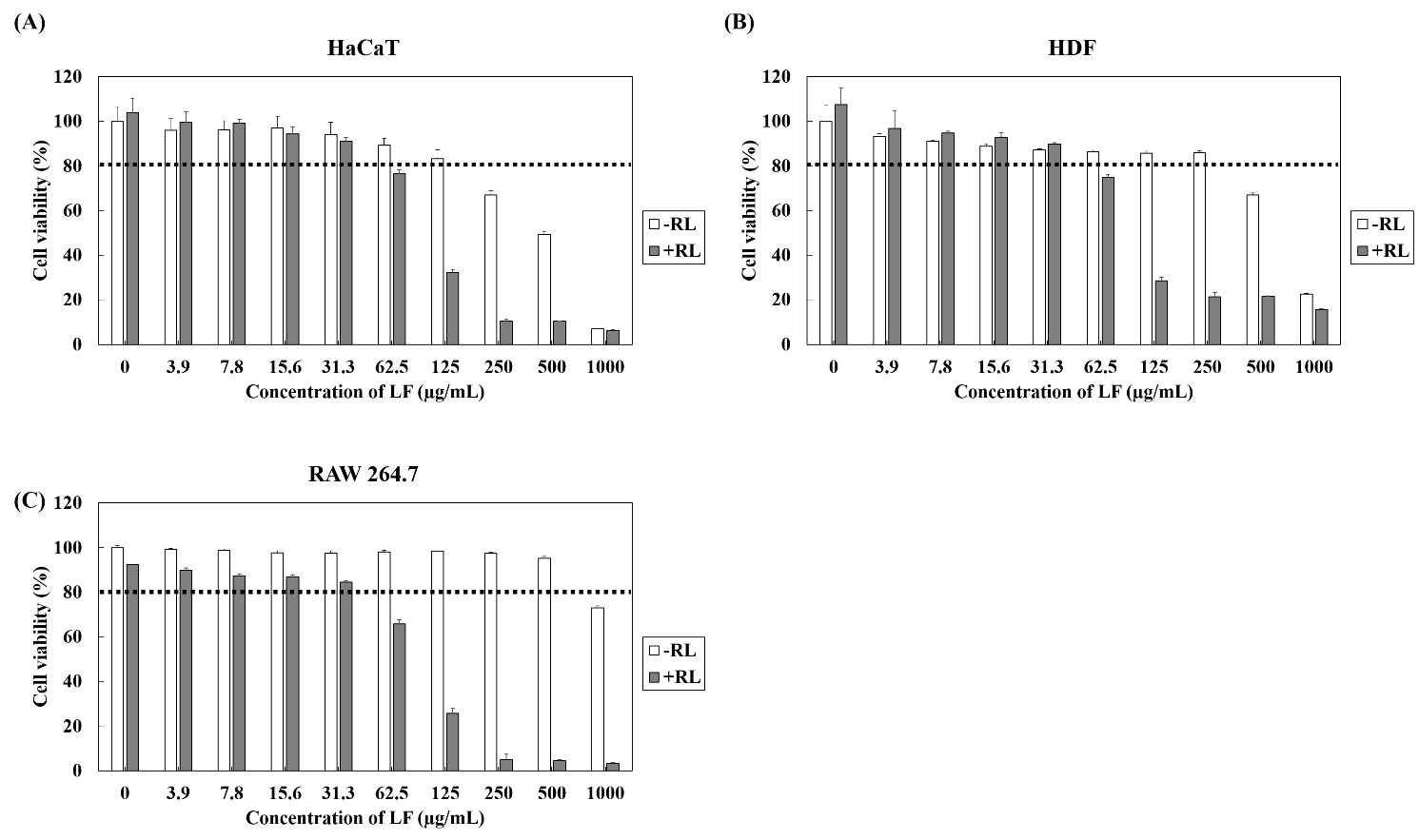


**FIGURE S4.** Cytotoxicity of LF with or without red light (RL) in HaCaT, HDF, and RAW 264.7 cells. Cell viability was assessed using an MTT assay. (A) HaCaT, (B) HDF, and (C) RAW 264.7 cells were treated with various concentrations of LF (0–1000 μg/mL) for 30 min in the dark, followed by RL irradiation. RL irradiation was performed using a red LED (660 nm, 120 W/m^2^, 15 min; light dose: 10.8 J/cm^2^). Cells were incubated for 24 h prior to viability measurement. Cell viability was normalized to untreated control cells (no LF, no RL), which were set to 100%. Data are presented as mean ± SD (n = 3). -RL, no irradiation; +RL, red light irradiation after LF treatment.


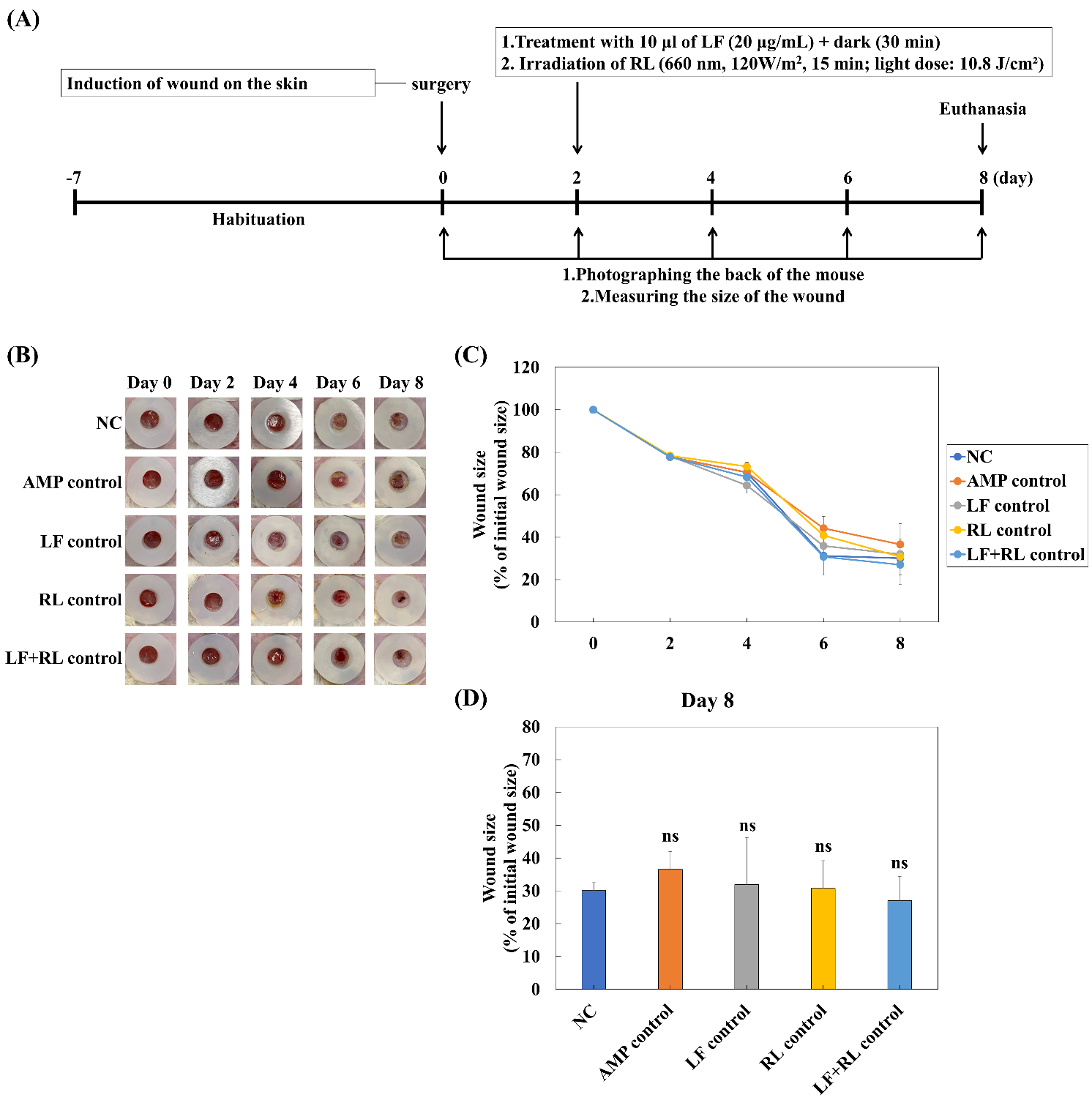
**FIGURE S5.** Effect of LF+RL on closure of acute wounds. (A) Schematic diagram of mouse splinted excisional wound model. (B) Representative images of wounds in different groups during experiment. Changes in wound size (C) over 8 days and (D) on day 8. Data are expressed as the mean ± standard deviation (n=6). ns, not significant; ^*^*p* < 0.05, ^**^*p* < 0.01, and ^***^*p* < 0.001 versus NC. Statistical significance is indicated at selected time points; non-significant differences are not shown for clarity. Statistical significance was analyzed by one-way ANOVA, followed by Tukey’s post-hoc test.


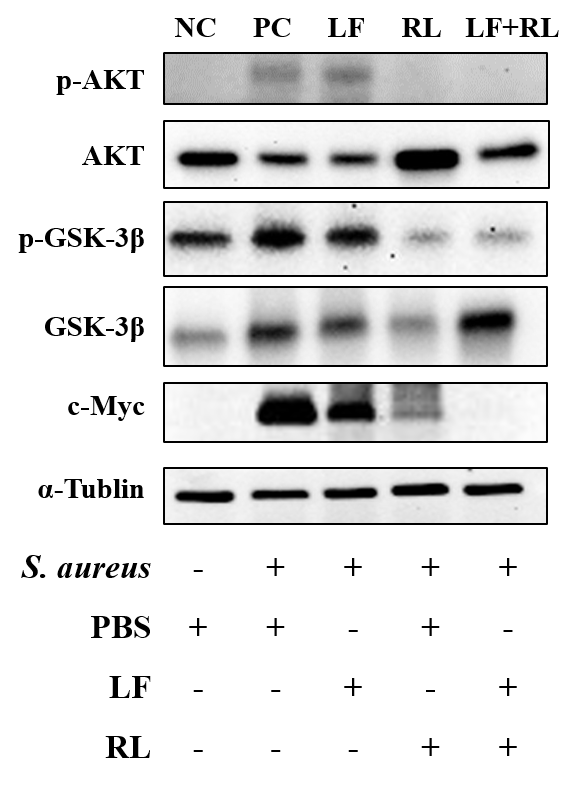


**FIGURE S6.** Effect of LF+RL on Akt/GSK-3β pathway. Western blot analysis was performed to determine phosphorylation of Akt and GSK-3β and expression of c-Myc.

**
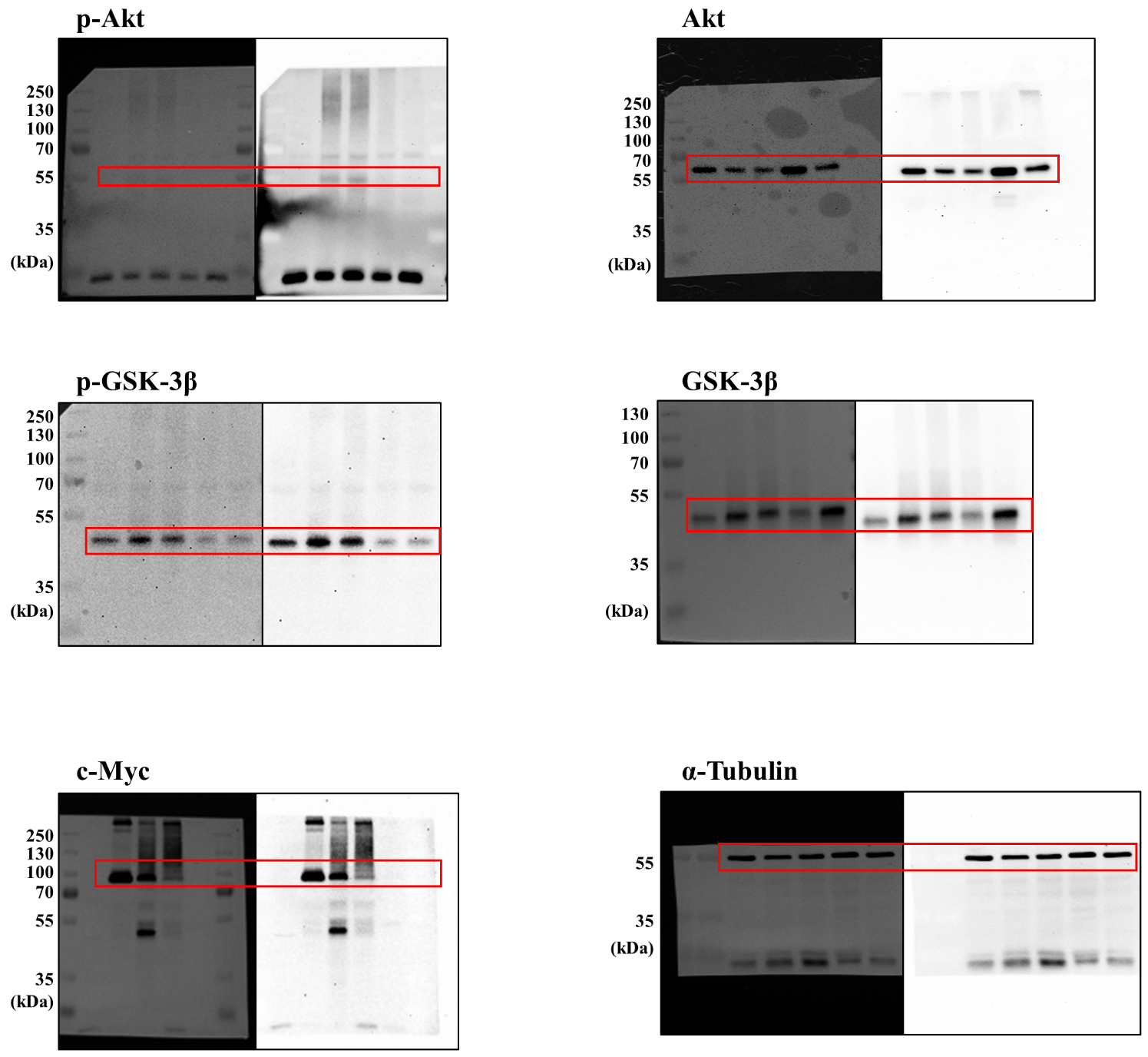
**

**FIGURE S7.** Full western blot membranes showing p-Akt, Akt, p-GSK-3β, GSK-3β, c-Myc, and α-tubulin.
